# A series of 4,5,9,10-tetrahydropyrene-based tetraarylethenes: synthesis, structures and solid-state emission behavior†

**DOI:** 10.1039/c8ra00057c

**Published:** 2018-04-24

**Authors:** Zhao-Ming Zhang, Bao-Xi Miao, Xin-Xue Tang, Zhong-Hai Ni

**Affiliations:** School of Chemical Engineering and Technology, China University of Mining and Technology Xuzhou 221116 People's Republic of China nizhonghai@cumt.edu.cn; Graduate School of Chemical Sciences and Engineering, Hokkaido University Sapporo 001-0021 Japan

## Abstract

By controlling the number of 4,5,9,10-tetrahydropyrene segments around the tetraarylethene core, a series of 4,5,9,10-tetrahydropyrene-based tetraarylethenes were synthesized and structurally characterized. An aggregation-induced emission (AIE) study indicated that all the compounds are AIE active: they are weak emitters in good solvents but highly emissive in the condensed phase, and hence are potential solid-state emitters. Their optical properties, electrochemical properties and theoretical calculations were investigated, and the results prove that the π-conjugation degree of these compounds increases with the increasing number of 4,5,9,10-tetrahydropyrene units. However, the fluorescence quantum yield in the solid state doesn't increase with increasing π-conjugation. We studied the reason for this by analyzing the crystal structures of some compounds, and proposed that the close degree of molecular packing in the solid state may be responsible for it. Loose packing of tetraarylethenes in the solid state can restrict the rotation of the aromatic rings but cannot constrain other non-radiative pathways efficiently, such as vibration, which leads to the unpredictable emission of the compounds.

## Abstract 2

Tetraarylethenes with different π-conjugation degrees can be realized by controlling the number of 4,5,9,10-tetrahydropyrene segments. However, the fluorescence quantum yield of these compounds in films doesn't increase with the π-conjugation degree.

## Graphical abstract

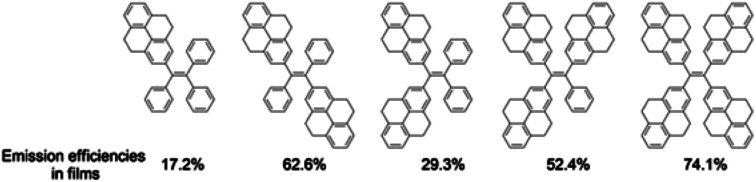

## 1. Introduction

The design and synthesis of organic materials with efficient solid-state fluorescence has attracted extensive attention because the performance of organic photoelectric devices is usually dependent on the solid-state properties of materials.^1–4^ And most luminogenic molecules, especially those with disc-like shapes, normally exhibit quenched emission in the condensed state due to the formation of intermolecular π-stacking interactions.^5–7^ Several strategies have been developed to solve this problem such as designing polymers with dendritic structures and using antiaggregation reagents to modulate aggregation.^8–10^ In these methods, the design ideas are obstructing the formation of luminophoric aggregates. Recently, it has been proved that the construction of organic compounds with aggregation-induced emission property is a convenient but effective way to obtain outstanding luminogens.^11–16^ Different from the ordinary organic compounds usually with excellent single-molecule emission in solvents, AIE compounds are weakly emissive in good solvent but they become strong emitters when the condensed phase are formed.^17^ In aggregates, intramolecular rotation which acts as a non-radiative pathway can be restricted effectively, meanwhile the twisted structure of these compounds can avoid the formation of π-stacking to a certain extent, thus achieving high solid-state emission efficiency. As a representative member of AIE dyes, tetraphenylethene (TPE) has attracted much research attention because it enjoys the advantages such as efficient solid-state emission, facile synthesis and easy functionalization.^18–20^ In addition, by combing TPE and many kinds of polycyclic aromatic hydrocarbons (PAHs), a large number of novel TPE derivatives have been developed.^21–27^ It has been reported that the fluorescence emission efficiency is affected by the π-conjugation length of compounds.^28^ In the cases of TPE derivatives, the introduction of PAHs can extend the π-conjugation of compounds and their good performance in emission suggests that enlarging the π-conjugation degree of AIE compounds is an effective strategy to obtain solid-state dyes. Based on this fact, we speculate that in solid state, intramolecular rotation of AIE compounds can be effectively restricted and π–π interactions are completely absent, whether the emission efficiency would increase with the increasing π-conjugation?

The best way to study this problem is to synthesize a series of PAHs-substituted tetraarylethenes and analyze their emission efficiency in solid state, in which the different π-conjugation degree can be realized by controlling the number of PAHs substituents. However, up to now, the reported PAHs-substituted arylethenes are concentrated on the tetraarylethenes with one and two PAHs moieties, but it is also necessary to obtain three and four PAHs-substituted tetraarylethenes in order to fully study the problem. Zhao *et al.* have successfully synthesized four naphthalene-substituted arylethene compounds from naphthalen-2-yltriphenylethene to tetra(naphthalen-2-yl)ethane.^29^ However, on account of the planar and bulk structure of the naphthalene, these four compounds suffered from the notorious π–π interactions, leading to a decrease tendency in emission efficiency with the increasing number of naphthalene substitution. Because of the interference of the π–π interactions, these naphthalene-based compounds cannot be employed to study the proposed problem directly. Therefore, a special PAHs is required urgently, based on which the constructed tetraarylethenes can avoid the formation of π–π interactions in solid state. In our previous work, we have reported that 4,5,9,10-tetrahydropyrene possesses both enlarged conjugation and certain steric hindrance, and the introduction of it into tetraarylethene system can expand the π-conjugation but prevent the formation of π–π interactions.^30,31^ Therefore, in this work, based on 4,5,9,10-tetrahydropyrene, a series of 4,5,9,10-tetrahydropyrene-based tetraarylethenes has been synthesized and their optical, electrochemistry properties and theoretical calculations have been fully investigated. Moreover, the effect of π-conjugation and molecule packing on solid-state emission has been discussed.

## 2. Experimental

### 2.1 General

All reagents were purchased from commercial sources as analytically pure grade. Tetrahydrofuran (THF) was distilled from sodium benzophenone ketyl under dry nitrogen immediately prior to use. Dichloromethane was dried and purified by distillation before use from CaH_2_. 4,5,9,10-Tetrahydropyrene was prepared by a method reported in the literature.^32^ The synthetic procedure and the detailed characterization of 2,2′-bis-(4,5,9,10-tetrahydropyrenyl)ketone and tetrakis(4,5,9,10- tetrahydropyren-2-yl)ethene have been reported in ref. 30.

^1^H and ^13^C NMR spectra were measured on a Bruker AV 400 spectrometer in CDCl_3_ using tetramethylsilane (TMS; *δ* = 0) as internal reference. Mass spectra were obtained on a JEOL JMS Q1000GC Mk II Quad GC/MS or a Bruker Ultraflextreme MALDI TOF/TOF mass spectrometer or an IonSpec HiResMALDI FT mass spectrometer. IR spectra were recorded on KBr pellets using a Nicolet 7199B FT/IR spectrophotometer in the region of 4000–400 cm^−1^. UV-Vis spectra were recorded on Shimadzu UV-3600 with a UV-VIS-NIR spectrophotometer. Emission spectra were performed on a Hitachi fluorimeter (F-4600). *Φ*_F_ values of the amorphous films were determined by FM-4P-TCSPC transient state fluorescence spectrometer using an integrating sphere. Fluorescence lifetimes were examined on an Edinburgh FS5 spectrofluorometer. Cyclic voltammetry experiments were performed with a CHI660E electrochemical work station using 0.1 M *n*-Bu_4_NPF_6_ in dichloromethane as supporting electrolyte. All measurements were carried out at room temperature with a conventional three-electrode configuration consisting of a platinum disk working electrode, a platinum auxiliary electrode and a calomel reference electrode. Single-crystal X-ray data of compounds 1 and 4 were collected on Bruker APEX II CCD diffractometer. The structures were solved by the direct method and refined by full-matrix least squares on *F*^2^. Hydrogen atoms were added geometrically and refined using a riding model. CCDC numbers for compounds 1 and 4 are 1548871 and 1548874, respectively.

### 2.2 Synthesis procedure and structural characterization

#### 2.2.1 Synthesis of 2-benzoyl-4,5,9,10-tetrahydropyrene

To a mixture of 4,5,9,10-tetrahydropyrene (1.03 g, 5 mmol), aluminum chloride (1.60 g, 12 mmol) and carbon disulfide (100 mL), a solution of benzoyl chloride (0.73 g, 5.1 mmol) in carbon disulfide (20 mL) was added dropwise at 0 °C under nitrogen atmosphere. After stirring for 30 min at 0 °C, the mixture was warmed to room temperature and stirred overnight. The solution was poured into ice and extracted with dichloromethane. The combined organic layers were washed with saturated brine solution and water, and dried over anhydrous magnesium sulfate. After filtration and solvent evaporation, the residue was purified by silica-gel column chromatography using hexane/dichloromethane as eluent. Pale yellow product was obtained in 68% yield. Mp: 117–119 °C. IR (KBr, cm^−1^): 3056, 2933, 2894, 2831, 1654, 1595, 1326. ^1^H NMR (400 MHz, CDCl_3_), *δ* (TMS, ppm): 7.89–7.83 (m, 2H), 7.66–7.59 (m, 1H), 7.58–7.49 (m, 4H), 7.26–7.29 (m, 1H), 7.18–7.11 (d, 2H), 2.95 (s, 8H). ^13^C NMR (400 MHz, CDCl_3_), *δ* (TMS, ppm): 196.64, 138.12, 136.19, 135.93, 135.28, 134.82, 132.12, 129.94, 129.85, 128.31, 128.23, 127.96, 126.17, 28.15. GC/EI-QMS: *m*/*z* 310 [M]^+^.

#### 2.2.2 Synthesis of (4,5,9,10-tetrahydropyren-2-yl)triphenylethene (1)

To a solution of diphenylmethane (0.45 g, 2.67 mmol) in dry tetrahydrofuran (10 mL) was added 0.9 mL of a 2.5 M solution of *n*-butyllithium in hexane (2.22 mmol) at 0 °C under an nitrogen atmosphere. The resulting orange-red solution was stirred for 30 min at that temperature. To this solution was added the 2-benzoyl-4,5,9,10-tetrahydropyrene (0.62 g, 2 mmol), and the reaction mixture was allowed to warm to room temperature with stirring during a 6 h period. The reaction was quenched with addition of an aqueous solution and the organic layer was extracted with dichloromethane and the combined organic layers were washed with saturated brine solution and dried over anhydrous magnesium sulfate. The solvent was evaporated and the resulting crude alcohol (containing excess diphenylmethane) was subjected to acid catalyzed dehydration as follows.

The crude alcohol intermediate was dissolved in about 20 mL of toluene in a 100 mL Schlenk flask fitted with a Dean–Stark trap. A catalytic amount of *p*-toluenesulfonic acid (0.076 g, 0.4 mmol) was added and the mixture was refluxed for 3 h and cooled to room temperature. The toluene layer was washed with 10% aqueous NaHCO_3_ solution and dried over anhydrous magnesium sulfate and evaporated to afford the crude compound 1. The crude product was purified by column chromatography on silica gel using hexane/dichloromethane as eluent. Compound 1 was obtained as a pale yellow solid in 82% yield. ^1^H NMR (400 MHz, CDCl_3_), *δ* (TMS, ppm): 7.25–7.04 (m, 18H), 6.76 (s, 2H), 2.88–2.79 (m, 4H), 2.74–2.64 (m, 4H). ^13^C NMR (400 MHz, CDCl_3_): 145.11, 143.96, 143.93, 143.51, 142.42, 141.16, 140.82, 135.33, 134.46, 131.46, 131.42, 131.27, 130.69, 128.97, 128.56, 128.40, 128.17, 127.86, 127.63, 127.59, 126.87, 126.34, 126.31, 125.90, 28.35, 28.12. MALDI-FT MS: *m*/*z* 460.2 [M]^+^.

#### 2.2.3 Synthesis of dis(4,5,9,10-tetrahydropyren-2-yl)-1,2-diphenylethene (2)

To a solution of 2,2′-bis-(4,5,9,10-tetrahydropyrenyl)ketone (0.62 g, 2 mmol), zinc dust (0.26 g, 4 mmol) in 80 mL dry THF was added dropwise titanium(iv) chloride (0.4 mL, 2 mmol) under nitrogen at −78 °C. After stirring for 20 min, the reaction mixture was warmed to room temperature and then heated to reflux for 12 h. The reaction mixture was cooled to room temperature and poured into water. The organic layer was extracted with dichloromethane and the combined organic layers were washed with saturated brine solution and water, and dried over anhydrous magnesium sulfate. After filtration and solvent evaporation, the residue was purified by silica-gel column chromatography using hexane/dichloromethane as eluent. Green solid of compound 2 was obtained in 62% yield. ^1^H NMR (400 MHz, CDCl_3_), *δ* (TMS, ppm): 7.23–7.02 (m, 16H), 6.80 (s, 4H), 2.88–2.79 (m, 4H), 2.74–2.64 (m, 4H). ^13^C NMR (400 MHz, CDCl_3_): 143.96, 142.57, 142.45, 140.98, 140.81, 135.30, 134.38, 131.50, 131.35, 130.70, 129.00, 128.85, 128.78, 128.71, 127.53, 126.79, 126.26, 125.86, 28.33, 28.09. MALDI-FT MS: *m*/*z* 588.3 [M]^+^.

#### 2.2.4 Synthesis of dis(4,5,9,10-tetrahydropyren-2-yl)-2,2-diphenylethene (3)

The procedure was analogous to that described for compound 1. Pale yellow of compound 3 was obtained in 34% yield. ^1^H NMR (400 MHz, CDCl_3_), *δ* (TMS, ppm): 7.21–6.99 (m, 16H), 6.80 (s, 4H), 2.88–2.78 (m, 4H), 2.74–2.64 (m, 4H). ^13^C NMR (400 MHz, CDCl_3_): 144.08, 143.48, 142.41, 141.30, 140.63, 135.30, 134.36, 131.30, 130.68, 129.00, 128.80, 128.53, 128.14, 127.52, 126.81, 126.19, 125.88, 28.34, 28.10. MALDI-FT MS: *m*/*z* 588.3 [M]^+^.

#### 2.2.5 Synthesis of tris(4,5,9,10-tetrahydropyren-2-yl)phenylethene (4)

The procedure was analogous to that described for compound 2. Yellow-green powder of compound 4 was obtained in 22% yield. ^1^H NMR (400 MHz, CDCl_3_), *δ* (TMS, ppm): 7.19–6.96 (m, 14H), 6.91–6.75 (d, 6H), 2.96–2.57 (m, 24H). ^13^C NMR (400 MHz, CDCl_3_): 142.37, 140.60, 135.33, 135.03, 134.38, 131.41, 130.76, 129.07, 128.91, 128.76, 128.66, 128.43, 127.75, 127.49, 126.77, 126.14, 125.87, 28.46, 28.15. MALDI-TOF MS: *m*/*z* 716.30 [M]^+^.

## 3. Results and discussion

### 3.1 Synthesis

**Scheme 1:**
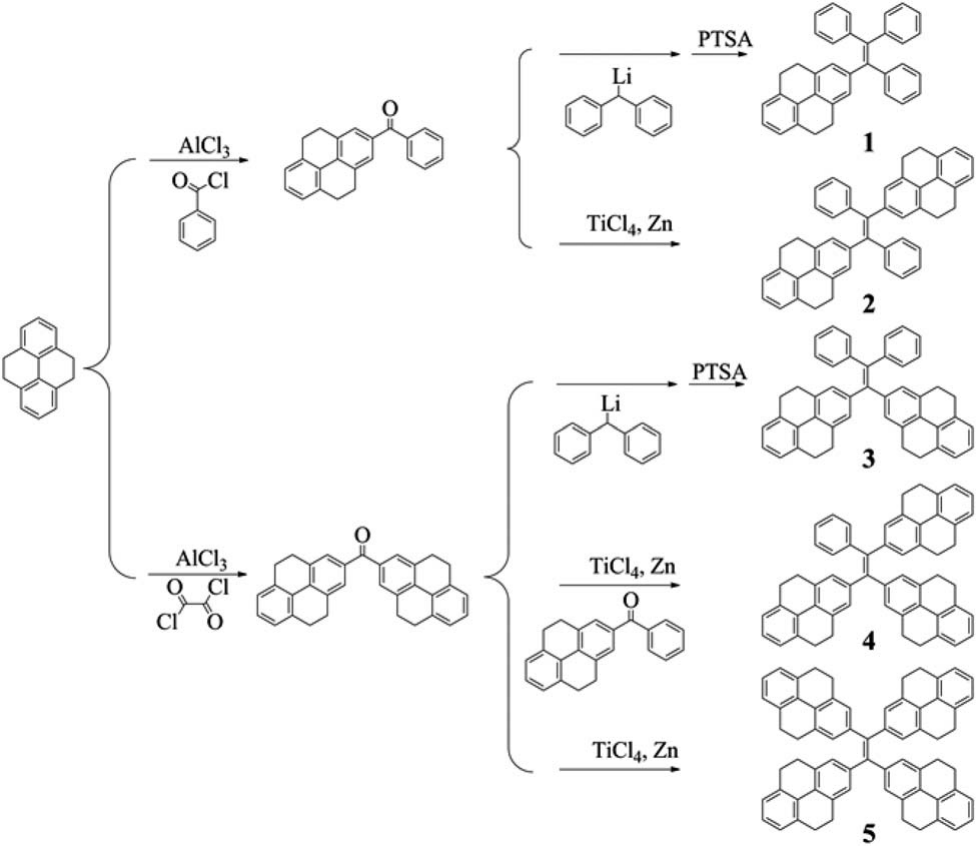
Synthetic routes to compounds 1–5.

Up to now, there are two main methods to synthesize tetraarylethenes. For asymmetric ethenes, Banerjee *et al.* proposed an effective method which is based on the reaction of diphenylmethyllithium and diaryl ketone.^33^ The other method is the famous McMurry coupling in which the diaryl ketone can be catalyzed by zinc powder and TiCl_4_ to afford the symmetric ethenes.^34,35^ In the two methods, it is obvious that the intermediate ketones play an important role in deciding the structures of the products. For the reported PAHs-substituted ethenes, ketones were usually obtained by the Friedel–Crafts acylation reaction of PAHs and benzoyl chloride. Based on these ketones, tetraarylethenes substituted by one and two PAH units can be synthesized very efficiently, for example, compounds 1 and 2 in this work were obtained with the yields of 82% and 62% (Scheme 1), respectively. However, the preparation of di(polycyclic aryl)ketones which are essential for the syntheses of tetraarylethenes with three or four PAH segments is difficult due to the limited synthetic methods. In our previous work, we have developed a convenient method in which PAHs can convert into their corresponding di(polycyclic aryl)ketones directly by one step reaction.^30^ Based on the McMurry coupling reaction of 2,2′-bis-(4,5,9,10-tetrahydropyrenyl)ketone and 2-benzoyl-4,5,9,10-tetrahydropyrene, tetraarylethene with three 4,5,9,10-tetrahydropyrene units can be obtained with relatively low yield because compounds 2 and 5 were also produced at the same time. Tetrakis(4,5,9,10-tetrahydropyrenyl)ethene can be conveniently obtained by the McMurry coupling reaction of pure 2,2′-bis-(4,5,9,10-tetrahydropyrenyl)ketone, which was reported in our previous work.^30^ In addition, a special molecule, compound 3 was also synthesized in this paper by the reaction of 2,2′-bis-(4,5,9,10-tetrahydropyrenyl)ketone and diphenylmethyllithium. To the best of our knowledge, this is the first tetraarylethene in which two PAH units are connected on one carbon and the other ethene carbon are substituted by two phenyl rings.

### 3.2 Optical properties

**Fig. 1:**
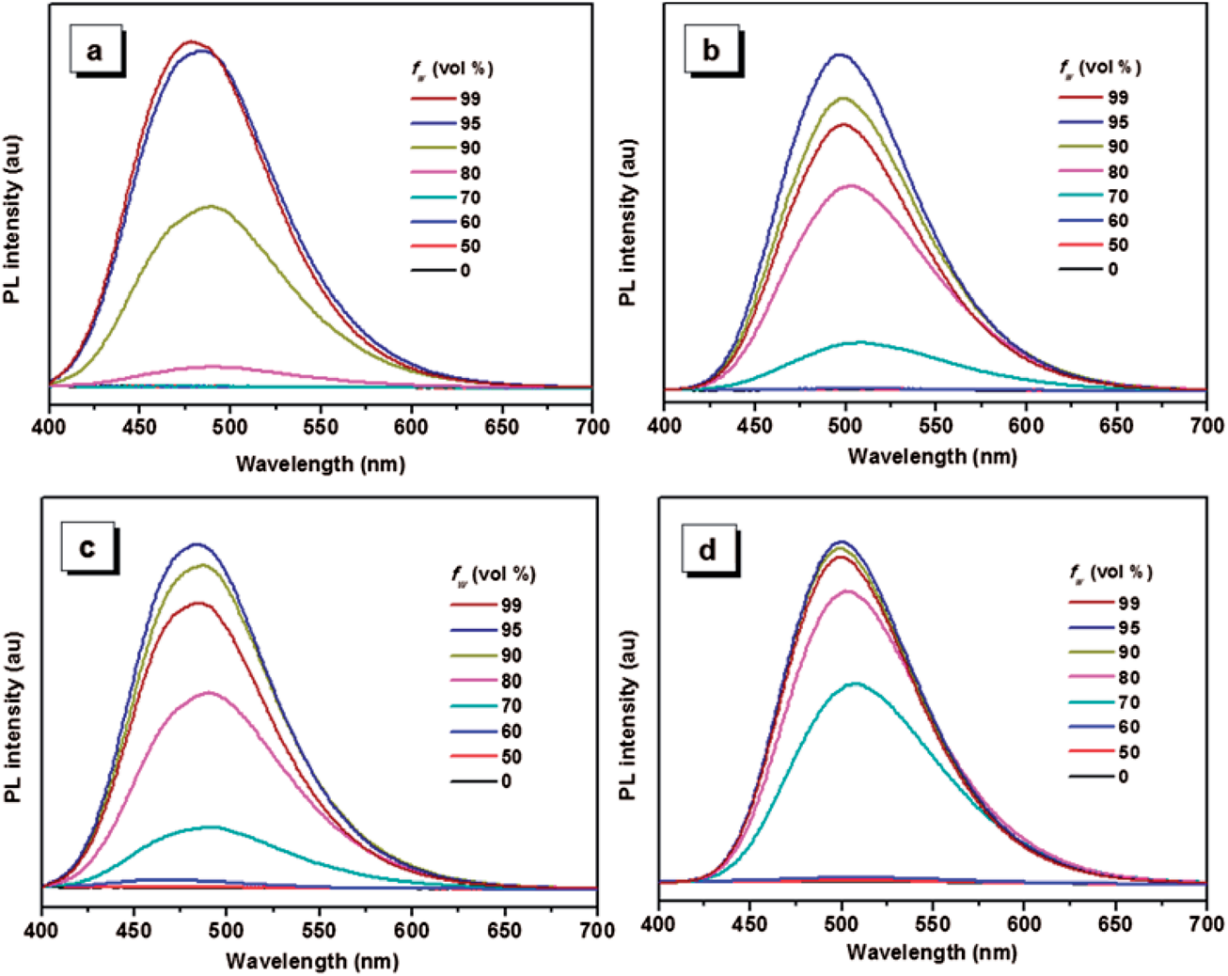
PL spectra of compounds 1 (a), 2 (b), 3 (c) and 4 (d) in THF/water mixtures with different water fraction (*f*_w_). Excitation wavelength: 360 nm.

The aggregation-induced emissions of compounds 1–4 were studied by monitoring the change in photoluminescence intensity with various composition of the THF/water mixture, which shows that all the five compounds are AIE active (the result of compound 5 see ref. 30). Taking compound 1 as an example (Fig. 1), the emissions are really weak when the water contents are lower than 80%, because the intramolecular rotation deactivates the excited state energy *via* a nonradiative decay pathway.^17,36^ Increasing the fraction of water to 80%, a peak located around 484 nm appears in the spectrum, and the emission intensity of this peak increase with the increasing water fraction until the 99% water content reaches the strongest emission. As a poor solvent, the addition of water into the THF solution induce the aggregation of the molecules, and the strong intermolecular interaction in aggregation can restrict the rotation of the aromatic rings, thus resulting in the strong emission. For the other compounds, same phenomenon can be observed when water was added into the THF solution. But compounds 2–5 show a decrease of emission efficiency in high water fraction, which has been well interpreted by many papers and it is ascribed to the formation of larger particles.^37^

**Fig. 2:**
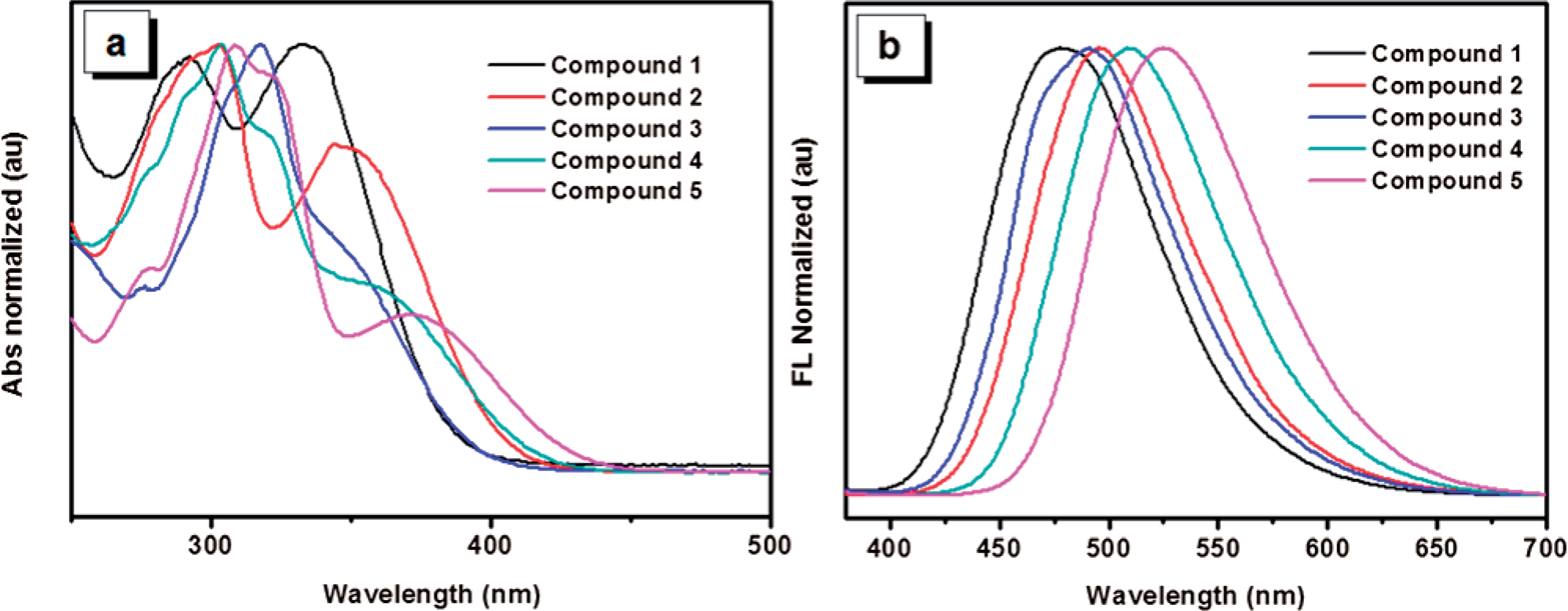
(a) UV-Vis spectrums of compounds 1–5 in THF solutions. (b) Photoluminescence spectrums of compounds 1–5 in films.

The UV-Vis spectra of the five compounds in THF solution are shown in Fig. 2a. Absorption maxima of compounds 1, 2 and 5 are observed at 334, 349 and 370 nm, respectively. Though accurate values of absorption maxima for compounds 3 and 4 are not so obvious, the tendency of the absorption maxima for the five compounds can be obtained basically with the order of 5 > 4 > 2 > 3 > 1. This order is consistent with that reflected by the optical band gaps which are derived from the onset of the absorption spectra for the five compounds (Table 1). The red shift of the UV spectra arising from the increasing number of 4,5,9,10-tetrahydropyrene units indicates that molecules having more 4,5,9,10-tetrahydropyrene units would possess a much stronger π-conjugation system. In addition, compared with compound 3, compound 2 has a red-shifted absorption, which suggests that though both of the two compounds have two 4,5,9,10-tetrahydropyrene moiety, the structure of compound 3 is not as effective as that of compound 2 to form a π-conjugation. As typical AIE compounds, these luminogens are weak emitters in dilute phase, so their emission behaviors in solution were not investigated in detail. However, different from the weak emission in solution, the solid films of these compounds exhibit intensive emissions. As shown in Fig. 2b, the corresponding emission peaks of compounds 1–5 appear at 480, 495, 491, 509 and 517 nm, respectively. It is noteworthy that the order of the emission wavelength is in good agreement with that of the absorption maxima, which further reveals the π-conjugation rule of the five compounds. Meanwhile, though some conformation change is possible due to the intermolecular interactions in aggregation, the emission of the films also indicates that the order of π-conjugation degree in film is similar with that in solution, which is very important to analyze their solid state emission in the following section.

**Table 1:** Optical and electrochemical properties of compounds 1–5

Compound	*λ*_abs_/nm	*λ*_em_/nm	*Φ*_F_ (%)	*E* ^ox^_onset_	HOMO^c^ (eV)		LUMO (eV)		*E*_g_ d (eV)	
	Solution^a^	Film^b^	Film		Expt.	Calc.	Expt.	Calc.	Expt.	Calc.
1	334	480	17.2%	1.16	−5.48	−5.122	−2.33	−1.299	3.15	3.823
2	349	495	62.6%	1.03	−5.35	−5.003	−2.41	−1.361	2.94	3.642
3	345	491	29.3%	1.04	−5.36	−5.005	−2.36	−1.356	3.00	3.649
4	355	509	52.4%	0.93	−5.25	−4.907	−2.46	−1.415	2.79	3.492
5	370	517	74.1%	0.82	−5.19	−4.823	−2.61	−1.435	2.58	3.388

a 1 × 10^−5^ M in THF solution.

b Film drop-casted on quartz plate.

c HOMO levels of compounds 1–4 were determined using the following equations: HOMO = −*e*(*E*_onset_ − 0.48 V) − 4.8 eV, where the value 0.48 V is for FOC *vs.* SCE electrode.

d Estimated from the onset of the absorption spectra: 1240/*λ*_onest_.

So far, it has been proved that all the five compounds are AIE active and thus their intramolecular rotation can be restricted effectively in solid state. Moreover, the twisted structures of the five compounds and the special structure of their 4,5,9,10-tetrahydropyrene moiety can avoid the formation of π–π interactions, which can be verified by the crystal packing structure discussed in later section. Therefore, it is reasonable to speculate that the emission efficiency of the five compounds in solid state would be decided by their π-conjugation with the order of 5 > 4 > 2 > 3 > 1.^28^ However, the real fluorescence quantum yields of their solid films which determined by using an integrating sphere don't obey this order and their values are 17.2%, 62.6%, 29.3%, 52.4% and 74.1% for compounds 1–5, respectively. We can find that though the π-conjugation of compounds 2 is not as good as compound 4, it has a higher value of fluorescence quantum yield in solid state. This problem is worthy to be studied because it ishelpful to reveal the mechanism of the solid-state emission and can provide reference for designing new solid-state emitter.

In order to have a deeper understanding of emission, the fluorescence lifetimes of the five compounds were also measured and the results are listed in Table 2. All these compounds show two different lifetimes, which indicates that there are two relaxation pathways in the process of their fluorescence decays. Therefore, it can be concluded that the emissions of the compounds arise from two kinds of segments with different π-conjugation lengths. For compounds 1, 2 and 5, the pathway of *τ*_2_ accounts for a larger proportion in the decay of the excited state. On the contrary, the compounds 3 and 4 mainly decay through the *τ*_1_ pathway. The weighted mean lifetimes of the five compounds are 2.71, 3.18, 2.00, 2.68 and 3.72 ns, respectively. Except for compound 1, there is a rule that the compounds which have higher emission efficiency tend to exhibit a longer fluorescence lifetimes. In addition, the fluorescence lifetimes of the compounds in THF solution were also measured, but no signal can be detected because of their AIE properties.

**Table 2:** Fluorescence decay parameters of compounds 1–5

Compound	*τ*_1_ (ns)	*A*_1_	*τ*_2_ (ns)	*A*_2_	〈*τ*〉 (ns)
1	1.38	0.46	3.85	0.54	2.71
2	1.43	0.41	4.40	0.59	3.18
3	1.47	0.81	4.27	0.19	2.00
4	1.65	0.59	4.15	0.41	2.68
5	1.48	0.36	4.98	0.64	3.72

### 3.3 Electrochemical studies

The electrochemical properties of the five compounds were studied by cyclic voltammetry (CV) in anhydrous dichloromethane, and corresponding data has been summarized in Table 1. The values of onset oxidation potentials (*E*^ox^_onset_) of the five compounds are 1.16, 1.03, 1.04, 0.93, 0.82 V, respectively, based on which the HOMO energy levels of these compounds have been calculated with the values of −5.48, −5.35, −5.36, −5.25 and −5.19 eV. Though these HOMO values are slight lower than those obtained by theoretical calculation, they still can reflect the information of π-conjugation of the five compounds with the order of 5 > 4 > 2 > 3 > 1 which is identical with the conclusion mentioned in the section of optical properties. The band gaps calculated based on the absorption edge in the absorption spectra are somewhat higher than those estimated by theoretical calculation, and their values are 3.15, 2.94, 3.00, 2.79 and 2.58 eV, respectively. The LUMO energy levels of the five compounds can be estimated by subtraction of the optical band gap energies from the HOMO energy levels with the values of −2.33, −2.41, −2.36, −2.46, and −2.61 eV, respectively.

### 3.4 Theoretical calculation

**Fig. 3:**
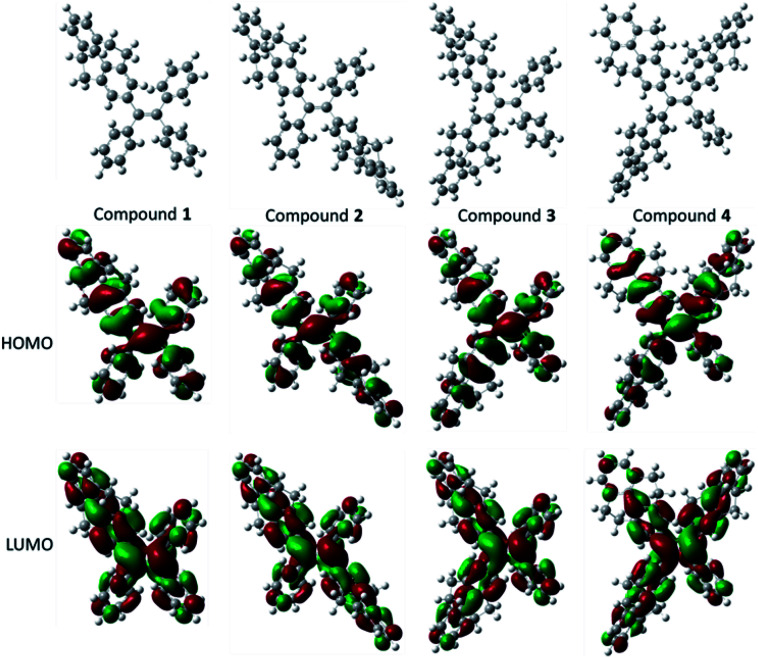
Optimized molecular structures, and molecular orbital amplitude plots of HOMO and LUMO of compounds 1–4.

The optical properties and electrochemical properties have disclosed the π-conjugation regularity of the compounds, and it is well known that the electronic structures can be employed to analyze the properties from a theoretical level. Therefore, density functional theory (DFT) calculations for the five compounds were carried out using a suite of Gaussian 09 program,^38^ and the nonlocal density functional of B3LYP with 6-31G(d) basis sets was used for the calculation. Fig. 3 displays the optimized molecular structures and orbital amplitude plots of HOMO and LUMO levels for compounds 1–4. It can be seen from the orbital amplitude plots that the HOMO and LUMO of five compounds (the result of compound 5 see ref. 30) are dominated by the orbitals from the aromatic rings as well as the central double carbon bond, indicating that the absorption and emission behaviors are associated with the π–π transitions and exciton decays of the whole molecules. In particular, both of the two phenyl rings in 4,5,9,10-tetrahydropyrene moiety contribute to the formation of the orbitals, which means that a 4,5,9,10-tetrahydropyrene can introduce larger π-conjugation than a phenyl moiety, and this result can be used to explain why the conjugation of the compounds increase with the number of 4,5,9,10-tetrahydropyrene moiety.

### 3.5 Crystal structure

**Fig. 4:**
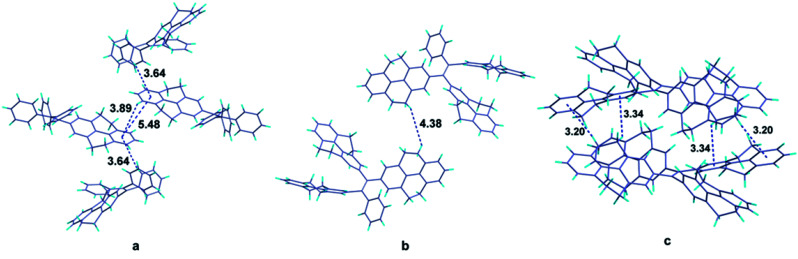
Packing structures of compound 1 (a), compound 4 (b), and compound 5 (c).

It is obvious that from the view of individual molecules, no reasonable interpretation can be found out to explain why the fluorescence quantum yields of the compounds don't have the same tendency with their π-conjugation. Fortunately, single crystals of compounds 1 and 4 as well as the reported compound 5 were grown from dichloromethane-methanol and analyzed by X-ray diffraction crystallography (Table S1†). The crystal structures of compounds can offer the information of intermolecular interactions, which are crucial to study the emission behaviors of compounds in solid state. In the crystal structure of compound 1 (Fig. 4a), the centroid distance of two phenyl rings in the 4,5,9,10-tetrahydropyrene moiety which belong to two adjacent molecules (the central two molecules in the figure), is 5.48 Å, which is much longer than the distance of typical π–π interactions. Except for these two phenyl rings, other aromatic rings are not likely to form π–π stacking. Therefore, we can draw a conclusion that π–π interactions are successfully avoided in the crystal structure of compound 1. Besides, the shortest CH/centroid distances observed in the crystal are 3.64 and 3.89 Å, which means that no strong C–H⋯π hydrogen bonds form in the crystal structure.^39,40^Fig. 4b is the crystal structure of compound 4. The packing of the molecules in the crystal structure are so loose that no potential π–π and C–H⋯π interactions can be observed. However, it has been reported that C–H⋯π hydrogen bonds can rigidify the molecular conformation and are beneficial to the emission of compounds.^41^ Considering the fact that compound 4 is AIE active, the rotation of the aromatic rings in solid state should be restricted by other weak intermolecular interactions such as van der Waals forces. But this kind of interaction is not enough strong to completely constrain the weak motion of the molecules like vibration which also can consume the excited energy in a non-radiative pathway. This may be the reason to explain why the emission efficiency of compound 4 is lower than that of compound 2.

Analyzing more crystal structures of TPE-based compounds, further evidence can be found to reveal the relationship between the degree of packing and their solid-state emission. In contrast with TPE, compound 1 has a much expanded π-conjugation system, whereas its solid-state fluorescence quantum yield (17.2%) is much lower than that of TPE whose value is 49.2%.^29^ From the view of packing, TPE has a much tighter stack in its crystal structure in which multiple C–H⋯π hydrogen bonds with short distances such as 3.047, 2.859 and 2.826 Å can be observed.^15^ It is noteworthy that similar with TPE, molecules of compound 5 in crystal state are also tightly stacked and numerous C–H⋯π interactions with distance of 3.20 and 3.34 Å are formed (Fig. 4c). Consequently, compound 5 also has good performance in its solid-state emission with 74.07% fluorescence quantum yield. Among these compounds, why TPE and compound 5 have a much tighter packing than others? Chemical structure of compounds is their intrinsic characteristic and is the decided factor for the packing. In TPE and compound 5, the ethenes are substituted by four same aromatic rings and thus they have a highly symmetrical structure, which should be more likely to form tightly packing. As for the higher emission efficiency of compound 2, though the crystal of compound 2 was failed to be obtained, it can be speculated that in solid state of compound 2, a much tighter packing may be formed compared with compound 4 because of its relatively symmetrical structure.

## 4. Conclusions

In conclusion, we have successfully synthesized a series of tetraarylethenes based on 4,5,9,10-tetrahydropyrene. These compounds are weakly fluorescent in solutions, but become strong emitters when the aggregations were formed, revealing distinct aggregation-induced emission characteristics. From the view of π-conjugation, corresponding characterizations including absorption and emission spectra, electrochemistry, and theoretical calculation support that the increase of 4,5,9,10-tetrahydropyrene moieties in compounds lead to the improvement of the π-conjugation with the order of 5 > 4 > 2 > 3 > 1. However, the fluorescence quantum yield of the compounds in films not fit the same order with the degree π-conjugation, and the value of compound 4 is not as high as that for compound 2. A reasonable interpretation can be found in the crystal structure of compound 4. Compared with other compounds, its crystal structure has a much looser packing. We speculated that the loose packing can restrict the rotation of the aromatic rings but cannot completely constrain other weakly non-radiative pathway such as vibration. Therefore, not only the properties of individual molecules but also their aggregates can affect the emission of compounds in solid state, and these two aspects need to be considered systematically when design new solid-state emitters.

## Conflicts of interest

There are no conflicts to declare.

## Supplementary Material

RA-008-C8RA00057C-s001

RA-008-C8RA00057C-s002
