# Supplementary material for: A series of 4,5,9,10-tetrahydropyrene-based tetraarylethenes: synthesis, structures and solid-state emission behavior
Source: RSC Adv. 2018 Apr 24;8(27):15173–80. doi: 10.1039/c8ra00057c (PMC9079984; doi:10.1039/c8ra00057c)
Supplement: RA-008-C8RA00057C-s001 [file RA-008-C8RA00057C-s001.pdf]

Electronic Supplementary Material (ESI) for RSC Advances.
This journal is © The Royal Society of Chemistry 2018

Supporting Information

A series of 4,5,9,10-tetrahydropyrene-based tetraarylethenes: synthesis, structures and solid-state emission behaviors

Zhaoming Zhang [a,b], Baoxi Miao [a], Xinxue Tang [a], Zhonghai Ni [a,*]

[a] School of Chemical Engineering and Technology, China University of Mining and Technology, Xuzhou 221116, People's Republic of China

[b] Graduate School of Chemical Sciences and Engineering, Hokkaido University, Sapporo 001-0021, Japan

* Corresponding author E-mail: nizhonghai@cumt.edu.cn; Tel: +86-516-83883927

Table S1

Crystal data and structure refinement parameters for compounds **1** and **4**

	Compound **1**	Compound **4**
Formula	$C_{35}H_{28}$	C_{56} H_{44}
Formula weight	460.58	716.91
Temperature (K)	293(2)	293(2)
Crystal system	monoclinic	triclinic
Space group	$P\ 2_1/c$	P -1
a (Å)	8.5141(17)	8.1823(16)
b (Å)	17.426(4)	12.694(3)
c (Å)	17.000(3)	19.994(4)
α(°)	90.0000(0)	90.14(3)
β(°)	97.44(3)	93.52(3)
γ(°)	90.0000(0)	101.80(3)
Volume (Å^3)	2500.9(9)	2028.7(7)
Z	4	2
ρ_{calcd} (g cm^{-3})	1.223	1.174
μ(Mo – Kα) (mm^{-1})	0.069	0.066
F(000)	976	760
Crystal size (mm)	0.10×0.08×0.06	0.08×0.08×0.06
Independent reflections	4387	7079
Reflections [$I > 2\sigma(I)$]	3540	4032
θ range (°)	3.19-25.00	3.16-25.00
Goodness-of-fit on F^2	1.091	1.051
$R_1[I > 2\sigma(I)]$	0.0832	0.0816
wR_2(all data)	0.2169	0.2150
